# Lymphoid stromal cells - potential implications for the pathogenesis of CVID

**DOI:** 10.3389/fimmu.2023.1122905

**Published:** 2023-02-17

**Authors:** Victoria N. Cousin, Guillermo F. Perez, Kathryn J. Payne, Reinhard E. Voll, Marta Rizzi, Christopher G. Mueller, Klaus Warnatz

**Affiliations:** ^1^ Department of Rheumatology and Clinical Immunology, Medical Center – University of Freiburg, Faculty of Medicine, University of Freiburg, Freiburg, Germany; ^2^ Center for Chronic Immunodeficiency (CCI), Medical Center – University of Freiburg, Faculty of Medicine, University of Freiburg, Freiburg, Germany; ^3^ University of Freiburg, Faculty of Biology, Freiburg, Germany; ^4^ Freiburg Spemann Graduate School of Biology and Medicine (SGBM), Albert Ludwigs University Freiburg, Faculty of Biology, Freiburg, Germany; ^5^ Immunologie, Immunopathologie et Chimie Thérapeutique, CNRS UPR3572, Strasbourg, France; ^6^ Faculty of Life Science, University of Strasbourg, Strasbourg, France; ^7^ Division of Clinical and Experimental Immunology, Institute of Immunology, Center of Pathophysiology, Infectiology and Immunology, Medical University of Vienna, Vienna, Austria

**Keywords:** lymphoid stromal cells, germinal center, common variable immunodeficiency, autoimmunity, stromal niche

## Abstract

Non-hematopoietic lymphoid stromal cells (LSC) maintain lymph node architecture and form niches allowing the migration, activation, and survival of immune cells. Depending on their localization in the lymph node, these cells display heterogeneous properties and secrete various factors supporting the different activities of the adaptive immune response. LSCs participate in the transport of antigen from the afferent lymph as well as in its delivery into the T and B cell zones and organize cell migration *via* niche-specific chemokines. While marginal reticular cells (MRC) are equipped for initial B-cell priming and T zone reticular cells (TRC) provide the matrix for T cell-dendritic cell interactions within the paracortex, germinal centers (GC) only form when both T- and B cells successfully interact at the T-B border and migrate within the B-cell follicle containing the follicular dendritic cell (FDC) network. Unlike most other LSCs, FDCs are capable of presenting antigen *via* complement receptors to B cells, which then differentiate within this niche and in proximity to T follicular helper (T_FH_) cells into memory and plasma cells. LSCs are also implicated in maintenance of peripheral immune tolerance. In mice, TRCs induce the alternative induction of regulatory T cells instead of T_FH_ cells by presenting tissue-restricted self-antigens to naïve CD4 T cells *via* MHC-II expression. This review explores potential implications of our current knowledge of LSC populations regarding the pathogenesis of humoral immunodeficiency and autoimmunity in patients with autoimmune disorders or common variable immunodeficiency (CVID), the most common form of primary immunodeficiency in humans.

## 1 Introduction

Stromal cells comprise of heterogeneous populations of mesenchymal cells, blood endothelial cells or lymphatic endothelial cells. The mesenchymal stromal cells differ by their tissue localization, their function and phenotype. LSCs are specialized stromal cell subpopulations within lymphoid tissues. They form reticular microenvironments to support adaptive immune cell retention, activation, proliferation, and differentiation in homeostatic conditions and in response to antigenic stimulation (1, 2). The reticular microenvironment of secondary lymphoid organs is mainly generated by heterogeneous fibroblastic reticular cells (FRCs) differing in length and number of cytoplasmic extensions. During an immune response, lymph nodes (LNs) require expansion to shelter proliferating lymphocytes. Thus, LSC plasticity is beneficial to accommodate LNs changes in size and architecture, including the formation of germinal centers (GC). FRCs and lymphatic endothelial cells (LEC), two LSC populations that express podoplanin, control this process of LN expansion. Binding of FRCs and LECs *via* podoplanin to C-type lectin-like type II transmembrane receptor (CLEC-2) on mature dendritic cells releases the lymph node internal tension and allows its expansion *via* FRC and LEC stretching and proliferation (3). LSCs create the niche properties of these microenvironments not only by structural components, but also by the secretion of soluble factors that guide, retain, and promote immune cells in specific zones of the lymph node. In a review published in 2021, Grasso et al. identified fourteen LSCs subsets in human and twenty-one in mice (4). Single-cell RNA-seq based experiments report up to nine different clusters of peripheral non-endothelial LSCs in mice (5).

Common variable Immunodeficiency (CVID) is the most common form of human primary immunodeficiency. The defining immunological feature is hypogammaglobulinemia and poor vaccine response (6, 7) due to impaired production of specific immunoglobulins especially of IgA, IgG isotype and reduced output of long-lived memory B cells and plasma cells (8, 9).

CVID comprises a heterogeneous group of disorders. CVID patients have been divided into subgroups based on either their clinical or their immunological presentation. When grouped according to clinical presentation, patients who present only with infectious complications belong to the CVIDio (infection-only) and patients who present further manifestations of immune dysregulation belong to the complex CVID (CVIDc) group. Immune dysregulation in CVIDc includes generalized lymphadenopathy, granulomatous disease, interstitial lung disease, gastroenteropathy and autoimmune manifestations (10). Additionally, different suggestions have been made how to divide CVID patients according to immunological parameters. The most commonly used classification is EUROClass (11, 12). Here, patients are divided based on the B-cell phenotype into patients with and without B cells. Patients with B cells are further subdivided into patients with normal or strongly reduced switched memory IgM-IgD-CD27+ B cells with or without an expansion of activated CD21low B cells (12). The more severe reduction of the switched memory B cells and the expansion of CD21low B cells is associated with the complex presentation of CVID patients (12). Both impaired production of specific class-switched immunoglobulins and reduced output of long-lived memory B cells and plasma cells point towards a disturbed function of GCs. Histologically, in some patients, GC formation is severely disturbed (13), in others GC formation is well preserved or even associated with follicular hyperplasia but associated with a failure of GC output (14). The CVIDc phenotype as well as the accumulation of CD21low B cells in peripheral blood is associated with the occurrence of ill-defined GCs in secondary lymphoid tissues (12). In 2014, Unger et al., described a perturbed organization of the FDC network in CVID patients with ill-defined GCs (15), but to this day, the potential implications of the lymphoid stromal compartment in CVID pathogenesis has never been characterized. Considering the coordinating role of stromal cells in the orchestration of the immune response, we propose a review of LSC subsets and their function and hypothesize potential detrimental effects of altered LSC-immune cell interaction in the failure of memory formation and immune tolerance in CVID.

## 2 Specialized stromal niches during an immune response

**Figure 1:**
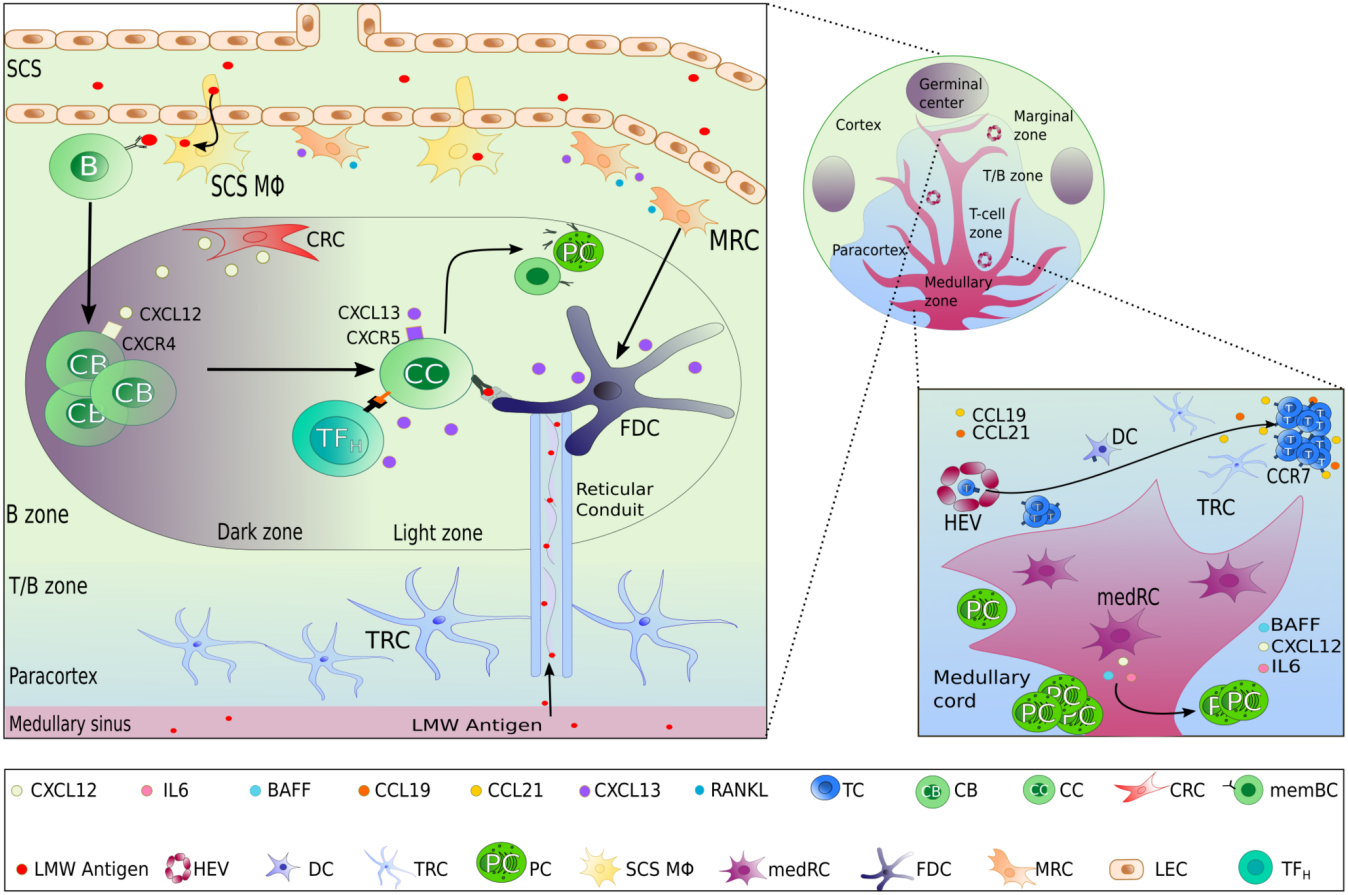
Lymph node stromal cells subset localization and functions. In the different zones of a lymph node, highly specialized lymphoid stromal cells (LSCs) secrete different chemokines and survival factors contributing to the organization of the lymph node, permitting the different steps of the adaptive immune response to take place successfully. Beside the selective secretion of chemokines, LSCs also contribute to the structure and transportation of lymph-derived antigens by the production of extracellular matrix including reticular conduits. FDC as the main LSCs within the light zone of GCs have the additional capacity of antigen presentation by surface expression of immune complex bound antigen.

Lymph nodes consist of the cortex, paracortex and medullar region, containing-B-cell follicles, T cells and plasma cells respectively (**Figure 1**). Afferent lymph drains into the subcapsular sinus where it encounters the first subset of stromal cells, the marginal reticular cells (MRC). MRCs produce CXCL13 and the receptor activator of nuclear factor kappa-B ligand (RANKL). MRCs reside in close proximity to LECs and subcapsular sinus macrophages, which present antigen to naïve GC precursor B cells to prime humoral immune response in GCs (16). In mice, MRCs generate a niche fostering the development of subcapsular sinus macrophages through RANK-RANKL interaction (17). MRCs are considered, along with CD21/Podoplanin double negative LSCs (18), as potential precursors of follicular dendritic cells (FDC), of which they can replenish after their depletion (19, 20). Interestingly, B-cell activating factor (BAFF), a significant hallmark of FDCs supporting B-cell differentiation and survival (21), is also expressed by MRCs (22). Additionally, Sato et al. identified splenic MRCs as phagocytes helping to clear apoptotic GC B cells in CD19eGFP mice, highlighting a central role of MRCs in GC homeostasis (23).

Chemokines and antigens contained in the lymph efficiently enter the paracortex, the area where T cells interact with antigen presenting cells (24), *via* reticular conduits assembled from extracellular matrix bundles of elastin or collagen (25, 26) secreted from by T zone reticular cells (TRCs). These conduits are found mainly within the paracortex and much less in the B-cell zone. TRCs control T-cell activation and localization in the paracortex by secreting CCL2 (ligand of CCR2), CCL5 (ligand of CCR1, CCR3 and CCR5), CCL7 (ligand of CCR1, CCR2, CCR3 and CCR5), and CXCL16 (ligand of CXCR6). TRCs also attract CCR7high-expressing B cells (27, 28) and T cells into the paracortex by secreting the CCR7 ligands CCL19 and CCL21 (**Figure 1**) (29). In addition to chemokine secretion, TRCs form guardrails with their cytoplasmic extensions, preventing T cells to migrate out of the T-cell zone. In 2014, Cremasco et al. performed a genetic ablation of lymph node TRCs in a mouse model and observed an altered localization of T cells in the paracortex (30).

At the initiation of an immune response, antigen-activated GC precursor B cells and activated T cells meet at the border of the T- and B-cell zone. In the case of a successful cognate interaction, both will progress to form a GC within B cell follicles of the cortex, where B cells undergo somatic hypermutation, affinity maturation, selection, and memory formation (31, 32). This highly specialized microenvironment consists of a “dark zone” and a “light zone” defined by the T-B cellular and cytokine composition. GC B cells usually migrate between both zones before differentiating into long-lived plasma or memory B cells (32). Both zones of the GCs present differences in reticular architecture and cellular diversity e.g. LSCs direct the formation of these zones by two opposite chemokine gradients: CXCL12 and CXCL13 (1, 33, 34). These chemokines diffuse in a solubilized form in the extracellular environment or alternatively, bind to extracellular matrix components to create short, immobilized gradients on extracellular surfaces (1, 35).

Within the dark zone, CXCL12-expressing reticular cells (CRCs) display thin and cytoplasmic extensions actively shaping a reticular network (34), allowing activated B cells, centroblasts, to crawl around the GC in response to a CXCL12 gradient. This is a result of CXCL12, also known as stromal-derived factor-1 (SDF-1), being the main ligand of the CXCR4 receptor, which is expressed on centroblasts (**Figure 1**). Interestingly, CRCs do not express the markers found on FDCs, FRCs, and complement 3-tagged (C3) antigen-capture mediators: FcɣRII, CD35, suggesting that CRCs do not act as antigen-presenting cells (34, 36).

**Figure 2:**
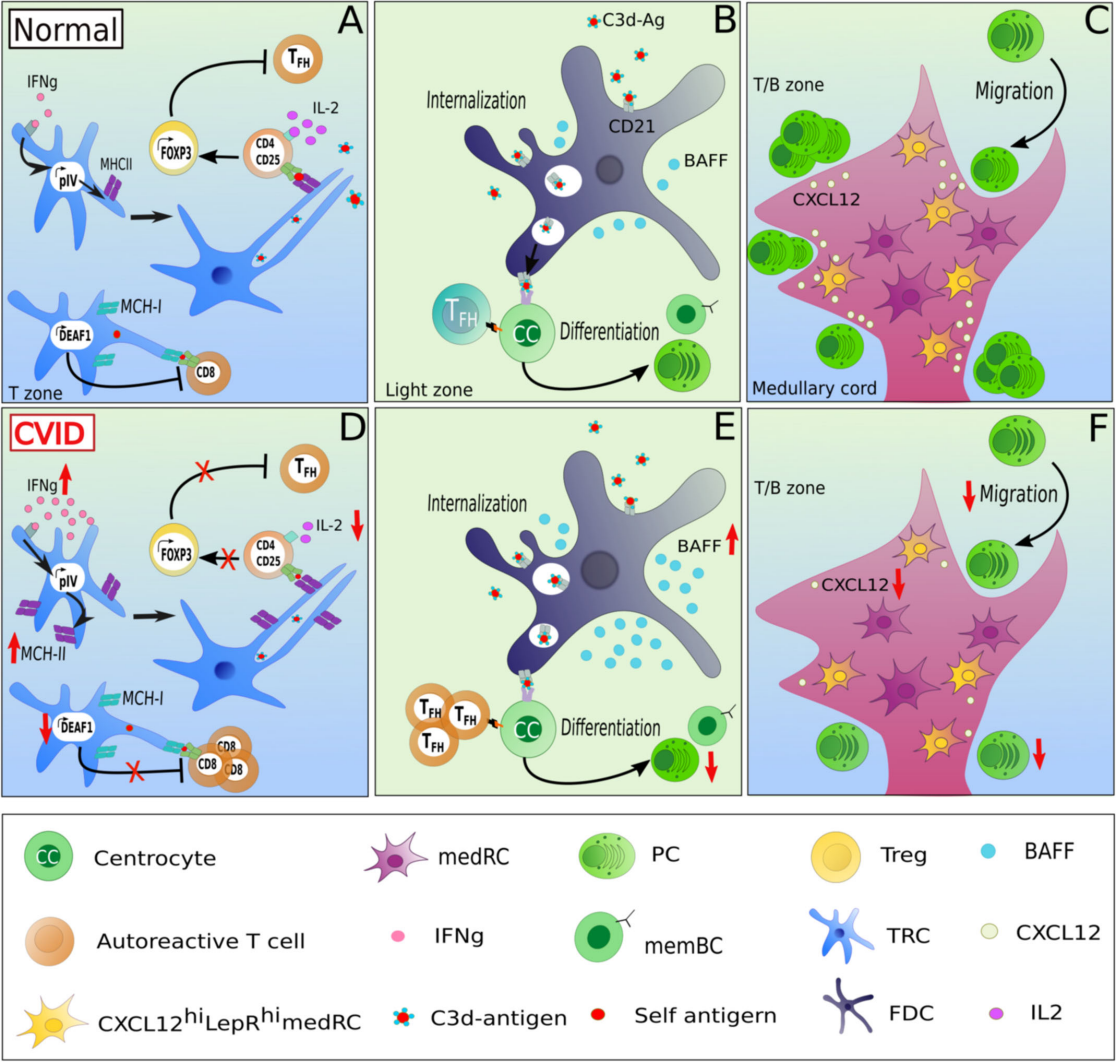
Potentially altered function of lymphoid stromal cells in patients with CVID and autoimmunity. LSCs may contribute to altered immune function in CVID by several mechanisms. In homeostasis, TRCs maintain tolerance through the presentation of self-restricted antigens *via* MHC-II expression, in an IFNɣ-dependant manner. IFNɣ activates the promoter IV region of the class II transactivator CIITA leading to MHC-II expression. Upon the recognition of self-restricted antigens presented by TRCs, CD4 T cells differentiate in an IL-2-dependent manner into FOXP3 T_reg_, preventing the expansion of autoreactive T_FH_ cells **(A)**. An increased MHC-II expression due to high IFNɣ expression in secondary lymphoid tissues combined with low local IL-2 levels could contribute to the expansion of T_FH_ cells at the expense of T_reg_ cells observed in lymph nodes of CVID patients with autoimmune manifestations **(D)**. Additionally, TRCs can also modulate tolerance of CD8 T cells by expressing DEAF-1, which mediates the a MHC-I mediated presentation of tissue-restricted antigens under tolerizing conditions thereby inhibiting the generation of autoreactive CD8 T cells **(A)**. A downregulation of DEAF1 in TRCs might contribute to the observed autoimmunity in some of the patients **(D)** In addition, as the serum of CVIDc patients often present high levels of BAFF, an increased production of BAFF by FDCs combined with a unlimited T_FH_ support increases the risk of a defective B cell selection **(B, E)**. A dampened CXCL12 secretion by CXCL12^hi^LepR^hi^ medRCs could alter the migration of CXCR4+ plasmablasts into the medullary cords **(C, F)** and contribute to the potential lack of GC-derived plasma cell response in CVID.

After proliferation and somatic hypermutation of BCR variable region genes of both heavy and light chain (31), centroblasts sequentially downregulate their CXCR4 expression and differentiate into non-proliferating CXCR5-expressing centrocytes and migrate towards the light zone. This movement occurs along the soluble homeostatic CXCL13 gradient, also known as B cell-attracting chemokine 1 (BCA-1) and is produced by FDCs and TFH in humans. CXCL13 can diffuse throughout the tissue reticular fibers (33); or alternatively, CXCL13 can interact with extracellular matrix components directly (such as heparin). This allows the formation of shortly and sharply immobilized CXCL13 gradients (1). CXCL13 is then able to re-solubilize *via* the activity of the protease cathepsin B, an essential process for the formation of B-cell follicle (1). FDCs create a network of cytoplasmic extensions in the light zone far denser than the CRC network seen in the dark zone. Centrocyte migration in the light zone is crucial during immune responses, BCR are selected for high affinity antigen receptor specificity by TFH cells and FDCs. FDCs, however, do not process antigens but acquire native antigens through immune-complex internalization *via* the complement receptor CR1 (CD35) or CR2 (CD21) in an actin-dependent manner. These immune complexes are retained in non-degrading endosomal vesicles for an extended period. Periodically, these complement-opsonized immune complexes cycle to the centrocytes cell surface (37) resulting in antigen-driven selection within the niche (**Figure 2**). Alternatively, FDCs can capture and retain lymph-borne low molecular weight antigens (smaller than 70kDa) that arise from reticular conduits unsheathed by TRCs cytoplasmic processes as mentioned above (**Figure 1**) (26, 38).

FDCs have been reported to support the survival and proliferation of positively selected centrocytes in GCs by the secretion of the survival factor BAFF (39, 40). On the other hand, Seyler et al. described that BAFF/APRIL blockade results in the destruction of the FDC network (41), indicating that BAFF is directly or indirectly involved in the formation or maintenance of the FDC network within GCs (42).

After positive selection in the light zone of GCs, centrocytes will differentiate into long-lived memory B cells or plasma cells (31, 32). While little is known whether there is a specific niche for memory B cells, plasma cells locate mainly to the medullary region of lymph nodes beside and before migrating to the bone marrow. In the lymph node, medullary reticular cells (medRC), and medullary macrophages, secrete interleukin-6 (IL-6), a cytokine involved in B cell differentiation into plasma cells, and survival factors such as the A-proliferation-inducing-ligand (APRIL), extending plasma cell survival (43, 44) (**Figure 1**).

Thus, the different subsets of LSCs play essential roles at all the sites of the normal immune response in secondary lymphoid tissues (**Table 1**).

**Table 1:** Lymphoid stromal cell subtypes in human.

Lymphoid Stromal Cell subtype	Abbreviation	Localization	Markers	Function
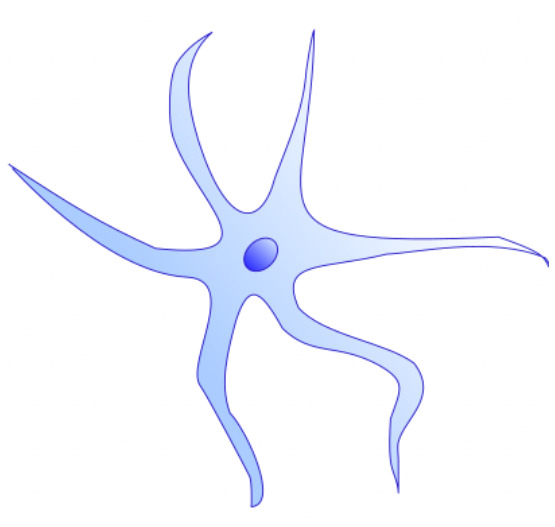	TRC: T-zone reticular cells	T zone	PDPN+ CD31- CCL21+ CCL19+ IL-7+, CD157+ Madcam1-	Shape reticular backbone of secondary lymphoid organs (SLO). Allow antigen circulation in reticular conduits. Produce collagen. Secrete chemotactic (CCL21, CCL19) and survival (IL-7) factors. Controls SLO microarchitecture through PDPN/CLEC-2 axis.
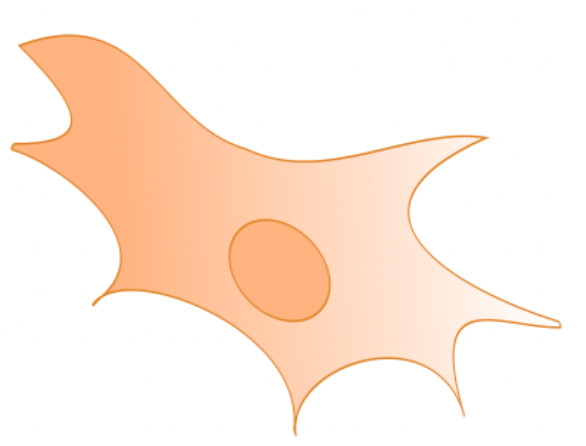	MRC: Marginal zone reticular cells	Marginal zone Interfollicular region. Below subcapsular sinus (SCS)	CD157+ Madcam1+	Precursors of FDC, secrete RANKL and CXCL13. Create a niche for SCS macrophages through RANKL expression
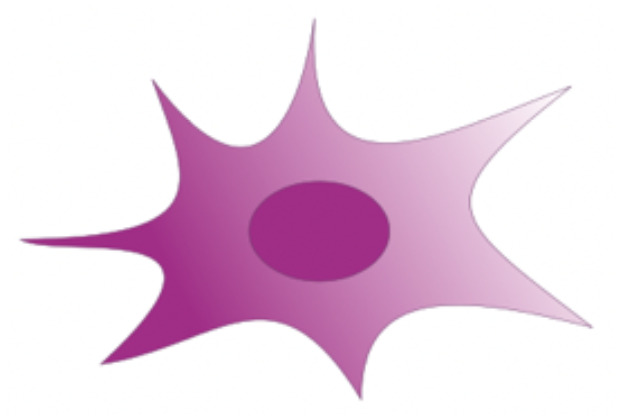	MedRC: edullary zone reticular cells	Medullary zone	CD157-Madcam1-	Secrete IL-6, CXCL12, BAFF. Form a dense meshwork inside medullary cords by expressing collagen I and laminin.
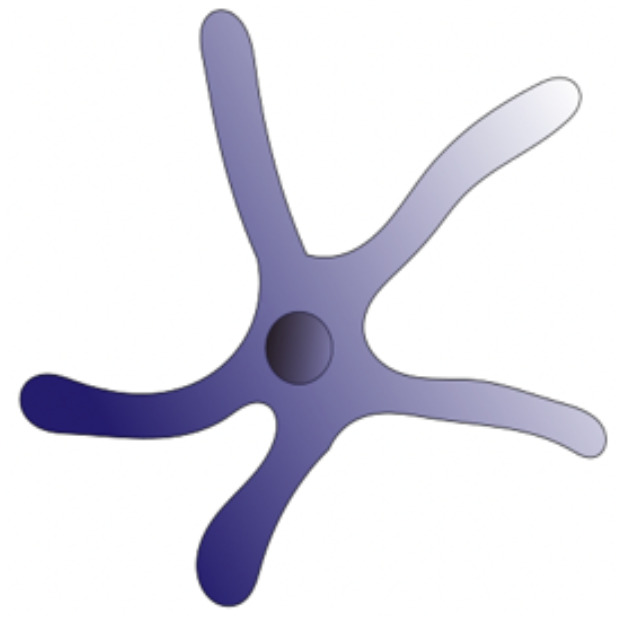	FDC: Follicular dendritic cells	Light zone of germinal centers (GC)	PDPN+ CD31- CXCL13+ CD21+ CD35+ BAFF	Immune complexes cycling and presentation through CD21 and CD35 expression. Secrete CXCL13 for B cell migration in the GC light zone. Shape a dense reticular network.
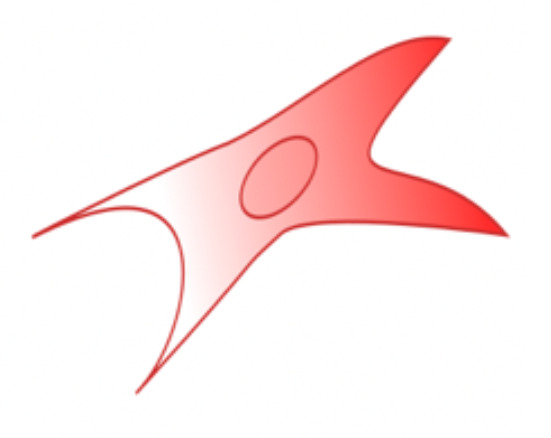	CRC: CXCL12 expressing reticular cells	Dark zone of GC	CXCL12+	Secrete CXCL12 for B cell migration in the GC dark zone. Shape a reticular network allowing B cells to easily crawl along CXCL12 gradient.

## 3 LSCs and loss of immune tolerance

LSCs are also involved in maintaining peripheral tolerance by regulating activation and expansion of autoreactive T- and B-cell clones within secondary lymphoid organs. This aspect of LSCs has mostly been studied in mice, with only a few studies describe these in human LSCs. In murine lymph nodes, FRCs and LECs mediate tolerance *via* the expression of the AIRE-like protein DEAF-1 (deformed epidermal autoregulatory factor-1 homolog), allowing the presentation of tissue-restricted antigens to CD8 T cells (45) (**Figure 2A**). These stromal cell subsets can act as analogs of medullary epithelial cells in the thymus mediating tolerance induction (45). In addition, LSCs can present tissue-restricted self-antigens *via* MHC-II to naive CD4 T cells in the absence of co-stimulatory molecules (46). The expression of MHC-II by TRCs is stimulated by the activity of the IFNγ-inducible promoter IV (pIV) region of the class II transactivator (CIITA) (**Figure 2A**) (46). The presentation of self antigens induces a conversion of CD4 T cells into FOXP3-expressing regulatory T cells (Treg) rather than autoreactive TFH cells in an IL-2/CD25 signaling-dependent manner (47). Blockade by neutralizing anti-IL-2 antibodies reduces Treg conversion and enhances autoreactive TFH cell proliferation, subsequently driving an expansion of autoreactive GC B cells (47) (**Figure 2B**). Additionally, deleting MHC-II in murine LECs leads to a reduced Treg proliferation, suggesting an essential role of LECs in supporting Treg homeostasis (48).

Furthermore, FDCs (**Figure 2B**) are a major source of IFNα and are thus considered as active players in the pathogenesis of murine SLE models (49). It is still unknown how an increased FDC proliferation induced by IFNα affects tolerance (50). Unfortunately, there is limited literature describing potential links between LSC function and SLE in humans. In the Mrl/lpr mouse model of lupus, lymph nodes display an abnormal architecture and hypertrophy (51). The expansion of autoreactive B cells in the 564 Igi mouse model of SLE (expressing a transgenic polyreactive BCR reacting to nucleic acid antigens), is promoted by IFNα secretion from FDCs after TLR7 stimulation by ssRNA. Thus, TLR-induced secretion of IFN type I by FDCs may contribute directly to an altered peripheral immune tolerance (49). Similarly, altered secretion of survival factors such as BAFF, in the GC niche can contribute to autoimmunity by extended survival of autoreactive cells as seen in a BAFF-transgenic mouse model (52) (**Figure 2B**).

In support of the reported role of stromal cells in tolerance maintenance or pathogenesis of autoimmunity (46, 48, 53–55), LSCs are thought to be involved in the pathogenesis of rheumatoid arthritis (RA). Indeed, the lymph node stromal microenvironment displays alterations during the earliest phases of the disease. *In vitro*, human RA LSCs express less CXCL12, and secrete less CCL19, CCL21 and CXCL13 upon stimulation with TNFα and LTα1β2 (56), potentially affecting migration, survival and selection of immune cells during the onset of RA (**Figure 2C**). Additionally, the capacity to expand lymph nodes during immune responses is also altered in RA-risk and in RA-early phase patients. RA LSCs display a reduced contractility as compared to non-disease LSCs (57). RA-risk individuals also show lower numbers of lymphoid tissue inducer cells, which are crucial for lymphoid tissue formation and homeostasis through close interactions with stromal cells (57, 58). Overall, these results suggest that the stromal compartment of lymph nodes is dysfunctional and potentially contributes to autoimmunity and RA.

## 4 Potential role of LSCs in the pathogenesis of common variable immunodeficiency (CVID)

The failure of long-lived switched memory and plasma cell responses in CVID (8) suggests a failure of GC function. Fitting this hypothesis, GC formation is frequently altered in CVID patient’s secondary lymphoid organs. Thus, in some patients mature GCs and plasma cells are absent in LNs, as seen in patients with ICOS (inducible co-stimulator) deficiency (13), while in LNs of CVID patients with non-malignant lymphoproliferation, GCs are gigantic but irregularly shaped, with plasma cells and memory B cells are still strongly reduced and displaced within follicles (15). Despite the fact that this knowledge exists for over 20 years, the pathomechanisms underlying the GC output failure is poorly understood. At present no report on LSCs - essential for establishing the GC niche - have been published in CVID patients. The only report on an altered stromal environment in CVID describes stromal cells isolated from bone marrow failing to support the development of pro-B cells into immature B cells (59). The alterations in secondary lymphoid tissues of CVID patients would provide unique opportunities to study the role and interaction of LSCs within the adaptive immune response. In 2014, Unger et al. showed by histological analysis a disruption of the CD23+ FDC network in addition to irregularly shaped gigantic and poorly polarized GCs in LNs of CVID patients (15). As LSCs are involved in shaping the GC compartment by the secretion of chemokines (CXCL12, CXCL13, CCL19, CCL21), survival factors (IL-7, RANKL), the generation of reticular conduits and the presentation of antigen (CR1, CR2 expression by FDCs), we hypothesize that an impaired interaction between lymphocytes and LSCs in CVID patients may contribute to the altered GC’s polarization and microenvironment and therefore result in the poor GC output in some of the patients. It is also intriguing that these irregularly-shaped GCs are associated with autoimmunity in these CVID patients characterized by an increased circulation of T-bethighCD21low B cells (15), an increased number of T-bet expressing cells in GCs, and local as well as systemic TH1-driven inflammatory environment (12, 60). Indeed, in CVIDc patients with immune dysregulation, the CD4 T cell differentiation appears to be redirected toward a TH1 phenotype. This shift leads to an accumulation of T-bethighCD21low B cells in peripheral blood and of increased IFNɣ production in secondary lymphoid organs (12) (**Figure 2D**).

As CVID patients with autoimmune manifestations often present a decreased TREG/TFH ratio (61), it will be interesting to test for a reduced IL-2 as an important factor regulating this ratio and a potentially altered capacity of FRCs and LECs to mediate tolerance by regulating autoreactive FH cell development of CD8 T cells as potentially suggested by Klocperk et al. (62) mediated by FRCs and LECs in CVID, the investigation of their expression of DEAF-1 in CVID-derived LN biopsies may be of interest (**Figure 2D**).

Up to 30% of CVID patients present autoimmune manifestations (11, 63, 64). These include different forms of autoimmunity ranging from mainly autoantibody-mediated, mixed T- and B-mediated autoimmunity in interstitial lung disease or arthritis and mostly T-cell driven autoimmune enteropathy. Autoantibody-mediated manifestations include especially autoimmune hemolytic anemia and immune thrombocytopenia (11) which represent the most frequent autoimmune manifestations in CVID. Autoimmune cytopenias are characterized by a reduced number of class-switched memory B cells, an expansion of CD21low B cells in peripheral blood (65, 66), as well as a dysregulated B and T cell homeostasis with elevated serum levels of Fas-ligand, IL-10 and BAFF (66). The best example for a T- and B cell mediated immune manifestation in CVID is the occurrence of a granulomatous and lymphocytic interstitial lung disease where Maglione et al. described even tertiary lymphoid structures containing B cells and CD23 positive FDCs within the affected lung tissue (67). Later, Ng et al. observed a dense and preserved CD21 positive FDC network in tertiary lymphoid structures of two CVID patients with interstitial lung disease (68). The bronchioalveolar fluids of these patients reflect the alveolar lymphocytic inflammation and contain a high percentage of CD21low B cells (69). Knowing that the stromal/immune cell interactions play a central role in maintenance of tertiary lymphoid structures by generating an environment favorable for lymphoid neogenesis (70), it would be of interest to investigate the stromal cell distribution and phenotype in TLS in lung biopsies from CD21low CVID patients with inflammatory or autoimmune pulmonary diseases.

The pro-survival factors of B cells and plasma cells BAFF and APRIL are often overexpressed in various autoimmune disorders including RA and SLE (41, 71). To date, mutations in their receptors BAFF-R (72) and TACI (73) and APRIL (74) but not BAFF have been associated with CVID. In 2007, Knight et al. reported high levels of BAFF and APRIL in peripheral blood of CVID patients (75). It remains to be investigated whether the autoimmune manifestations observed in some CVID patients are associated with BAFF and APRIL increased concentrations. Knowing that dendritic cells, macrophages (76), FDCs (77, 78), medRCs (44) and possibly MRCs (79) can secrete BAFF/APRIL, high concentrations of BAFF/APRIL in peripheral blood may reflect altered BAFF/APRIL secretion by the stroma niche of GCs, supporting the local expansion and survival of autoreactive B cells in CVID patients with autoimmune dysregulations (**Figure 2E**). Similarly, the displacement of class-switched plasma cells to intra-follicular instead of medullary regions in many CVIDc patients with lymphadenopathy (15) may be secondary to altered LSC mediated chemokine gradients or plasma cell niches (**Figure 2F**). In 2018, Takeuchi et al. described a medRC subset located in the medullary cords of mice (80). These cells were CXCL12highLepRhigh, and thus might be involved in the generation of the CXCL12 gradient between the paracortex and the medulla, allowing the migration of CXCR4 positive plasmablasts in medullary cords. Assessing CXCL12 production and expression in medRCs of CVID patients might bring new insights in understanding the potential defective migration of CXCR4 positive plasmablasts to medullary cords (**Figure 2F**).

The investigation of altered shape, size and disturbed function of GCs and plasma cell niches within secondary lymphoid tissues of patients with immunodeficiency and immune dysregulation like CVID or specific molecularly defined immunodeficiencies, provide a unique opportunity for in depth characterization of human lymph node stromal environment by novel technologies like single cell investigation and high-resolution immunofluorescence. These findings will not only provide key insights into the dysregulation of GC function in CVID but will potentially influence our understanding of factors relevant in peripheral immune tolerance as well as vaccine response.

## Data availability statement

The original contributions presented in the study are included in the article/supplementary material. Further inquiries can be directed to the corresponding author.

## Author contributions

VC, CM, and KW conceived the design of the review and hypothesis in discussion with all coauthors. VC wrote the first draft of the manuscript. GP, and CM wrote sections of the manuscript. All authors contributed to the article and approved the submitted version.
